# Achieving large linear elasticity and high strength in bulk nanocompsite via synergistic effect

**DOI:** 10.1038/srep08892

**Published:** 2015-03-09

**Authors:** Shijie Hao, Lishan Cui, Fangmin Guo, Yinong Liu, Xiaobin Shi, Daqiang Jiang, Dennis E. Brown, Yang Ren

**Affiliations:** 1 State Key Laboratory of Heavy Oil Processing, China University of Petroleum, Beijing 102249, China; 2 School of Mechanical and Chemical Engineering, The University of Western Australia, Crawley, WA 6009, Australia; 3 Department of Physics, Northern Illinois University, De Kalb, Illinois 60115, USA; 4 X-ray Science Division, Advanced Photon Source, Argonne National Laboratory, Argonne, Illinois 60439, USA

## Abstract

Elastic strain in bulk metallic materials is usually limited to only a fraction of 1%. Developing bulk metallic materials showing large linear elasticity and high strength has proven to be difficult. Here, based on the synergistic effect between nanowires and orientated martensite NiTi shape memory alloy, we developed an *in-situ* Nb nanowires -orientated martensitic NiTi matrix composite showing an ultra-large linear elastic strain of 4% and an ultrahigh yield strength of 1.8 GPa. This material also has a high mechanical energy storage efficiency of 96% and a high energy storage density of 36 J/cm^3^ that is almost one order of larger than that of spring steel. It is demonstrated that the synergistic effect allows the exceptional mechanical properties of nanowires to be harvested at macro scale and the mechanical properties of matrix to be greatly improved, resulting in these superior properties. This study provides new avenues for developing advanced composites with superior properties by using effective synergistic effect between components.

The quest for efficient energy-related technologies requires high-performance materials with large linear elasticity and high yield strength, which are important for mechanical energy storage, miniaturization and simplification of devices. However, the elastic strain in bulk metallic materials is usually limited to only a fraction of 1% due to the initiation of dislocation or twin activities when the applied stress reaches a critical value. Although many shape-memory alloys, such as NiTi and CuZnAl, can show large recoverable strains greater than several percent^1^,^2^,^3^, they behave non-linear and have large hysteresis^4^,^5^,^6^. To date, much effort has been made to develop bulk metallic materials with large linear elasticity and high strength, but the success has been very limited^7^,^8^,^9^,^10^,^11^,^12^.

It has been reported that the NiTi alloy with oriented martensitic variants, formed by martensite reorientation, exhibit large quasi-linear elasticity with 3–4% recoverable strains^7^,^8^, which consists of an elastic strain and a reversible twinning-detwinning strain^7^,^8^,^12^. The reason for such quasi-linear elasticity, rather than linear elasticity, is the reversible twinning-detwinning deformation, which dissipates mechanical energy causing a large hysteresis in its tensile cyclic curve. Owing to this, it is expected that the large hysteresis should be reduced or even eliminated, if the elastic deformation without energy dissipation can be greatly enhanced and the twinning-detwinning deformation with energy dissipation can be significantly decreased during tensile cycling by using an ingenious design.

It is known that freestanding nanowires have ultra-large elastic strains (4–7%) and ultrahigh yield strengths (often well above 1 GPa)^13^,^14^,^15^,^16^,^17^,^18^,^19^. Given this, much effort has been made in the past decade to create bulk composites of superior mechanical properties by enforcing them with nanowires. However, outcomes of most such attempts have been less than satisfactory, primarily because the exceptional intrinsic mechanical properties of nanowires cannot be exploited in their composites^20^,^21^,^22^. Until recently, based on a new principle of lattice strain matching between embedded nanowires and matrix, the exceptional mechanical properties of nanowires were exploited in a martensitic transforming metal matrix deforming by collective atomic migration without dislocation slip^23^. Given that both elastic deformation and twinning-detwinning deformation are also collective atomic migration without dislocation slip^24^,^25^, it is expected that the exceptional intrinsic mechanical properties of nanowires may be exploited in the metal matrix deforming by elastic elongation and/or twinning-detwinning. On the other hand, the embedded nanowires may also contribute to refining grain size of the matrix and introducing large amounts of nanowire/matrix interfaces, thus the elastic strain and yield strength of matrix could be significantly enhanced. Based on the above synergistic effect between nanowires and matrix, we expected that the composite made with nanowires and NiTi of oriented martensite variants may exhibit large linear elasticity and high strength.

In this study, we selected the NiTi of oriented martensite variants as a matrix to be combined with Nb nanowires. The NiTi-Nb system with ~10 atomic % Nb undergoes hypo-eutectic solidification into a microstructure consisting of fine Nb lamellae^26^, which can be converted into Nb nanowires through severe plastic deformation^27^. It is suggested the *in-situ* Nb nanowires-NiTi matrix composite simultaneously possesses ultra-large linear elastic strain of 4%, high yield strength of 1.8 GPa, high mechanical energy storage density of 36 J/cm^3^ and high energy storage efficiency of 96%, stemming from the synergistic effect between nanowires and matrix.

## Results and Discussion

**Figure 1:**
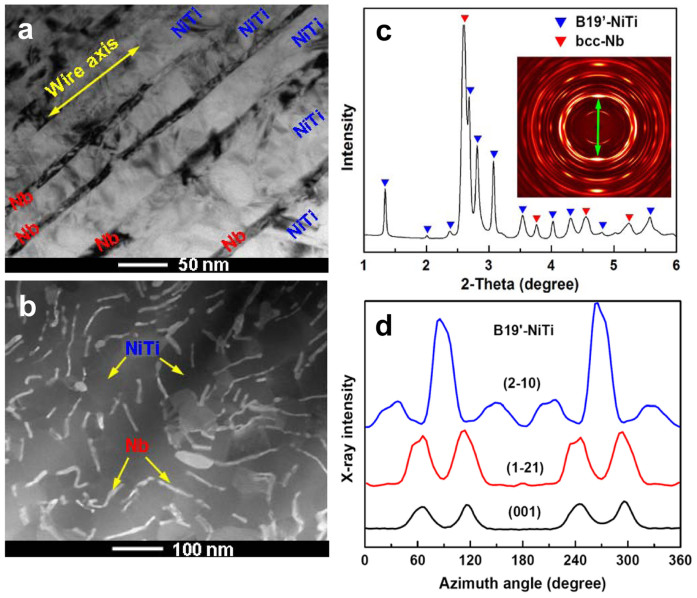
Typical microstructure of *in-situ* Nb nanowires -oriented martensitic NiTi matrix composite wire. (a–b), Scanning transmission electron microscopic (STEM) image of the longitudinal-section and cross-section of the composite wire (bright regions: cross sections of Nb nanowires; dark regions: NiTi matrix). (c), 1D high-energy X-ray diffraction (HE-XRD) pattern. Inset is its corresponding 2D HE-XRD pattern. Green arrow represents the wire axial direction. (d), Evolution of HE-XRD intensity for multiple planes of B19′-NiTi phase along the Debye-Scherrer rings recorded on area detector diffraction image (inset of Figure 1c).

An ingot with a composition of Ni_45_Ti_45_Nb_10_ was prepared by means of vacuum induction melting and casting. Macroscopic wire of the *in situ* composite was subsequently fabricated by forging, wire-drawing, annealing and pre-deformation treatment (Experimental Section for its fabrication details). The microstructure of the composite wire is shown in Figures 1a–b. The ribbon-shaped Nb nanowires, 5–20 nm in thickness and 40–200 nm in width, are well-dispersed and well-aligned in the NiTi matrix along the wire axial direction. The volume fraction of Nb nanowires is about 12%. The 1D high-energy X-ray diffraction (HE-XRD) pattern (Figure 1c) can be indexed to body-centered cubic Nb and B19′-NiTi phases. The 2D HE-XRD pattern (inset of Figure 1c) indicates that the Nb nanowires are well oriented in the [110] direction parallel to the wire axial direction. The evolution of the intensity for multiple planes of B19′-NiTi phase along the Debye-Scherrer rings recorded on area detector diffraction image (inset of Figure 1c) is shown in Figure 1d, the uneven distribution of diffraction intensity along the Debye-Scherrer rings indicates that the martensitic NiTi matrix is composed of oriented martensitic variants^28^.

**Figure 2:**
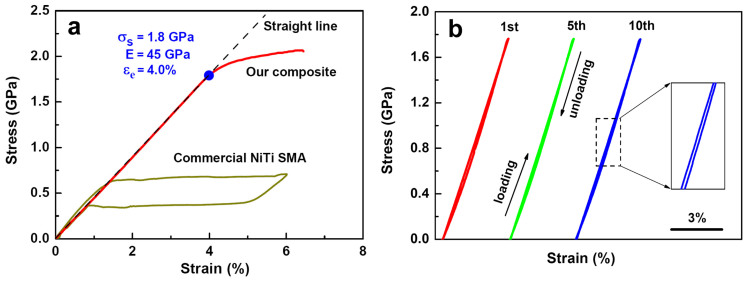
Mechanical properties of *in-situ* Nb nanowires - oriented martensitic NiTi matrix composite wire. (a), Comparison of tensile stress-strain curves of the composite wire (red curve) and a commercial superelastic NiTi wire (yellow curve). The black dotted line is a standard straight line. (b), Repeated cyclic tensile stress-strain curves of the composite wire. Inset is the enlarged view of the tensile cyclic curve.

Figure 2a shows the tensile stress-strain curve of the composite wire. For comparison, the tensile stress-strain curve of a commercial superelastic NiTi wire (Ni-49.4 at.% Ti) is also presented in Figure 2a. It is evident that the composite wire exhibits an ultra-large linear elastic strain of 4% and high yield strength of 1.8 GPa. The linear elastic strain limits about one order of magnitude higher than that of conventional bulk metallic materials. Figure 2b shows the cyclic tensile stress-strain curves of the composite wire. It is observed that the tensile loading and unloading curves are nearly fully over lapped and that the composite wire has an excellent cyclic stability. Moreover, comparisons of the tensile cyclic curves of our composite and the previous reported bulk metallic materials with large linear elasticity^7^,^8^,^9^,^10^,^11^,^12^,^23^. It is apparent that the hysteresis of our composite is much smaller than those of reported metallic materials.

**Figure 3:**
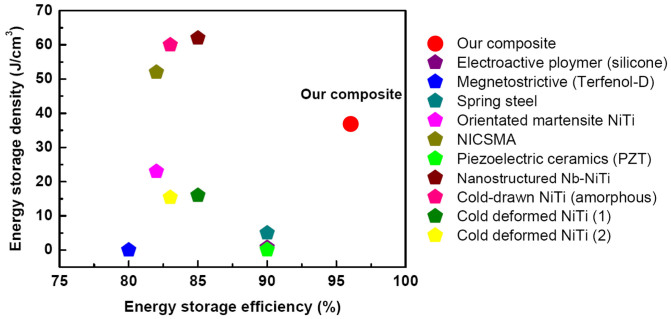
Comparison of mechanical energy storage density and storage efficiency between the *in-situ* Nb nanowires - oriented martensite NiTi matrix composite and other various advanced materials^7^,^8^,^9^,^10^,^11^,^12^,^23^.

Owing to the large linear elastic strain and high yield strength, the composite possesses a high mechanical energy storage density (the area under the tensile loading curve) of 36 J/cm^3^, which is almost one order of magnitude larger than that of spring steel (~5 J/cm^3^)^11^, and a high energy storage efficiency (the ratio of areas under the loading and unloading curves) of 96% in a tensile deformation cycle up to 4% of strain. Comparisons of mechanical energy storage density and storage efficiency of our composite and various other advanced materials are shown in Figure 3 and Table S1^7^,^8^,^9^,^10^,^11^,^12^,^23^. It is apparent that our composite occupies a unique position on the chart of mechanical energy storage density vs. energy storage efficiency. The unique combination of high energy storage density and high energy storage efficiency renders the composite great potential for application as novel mechanical energy storage and conversion materials with high efficiency.

**Figure 4:**
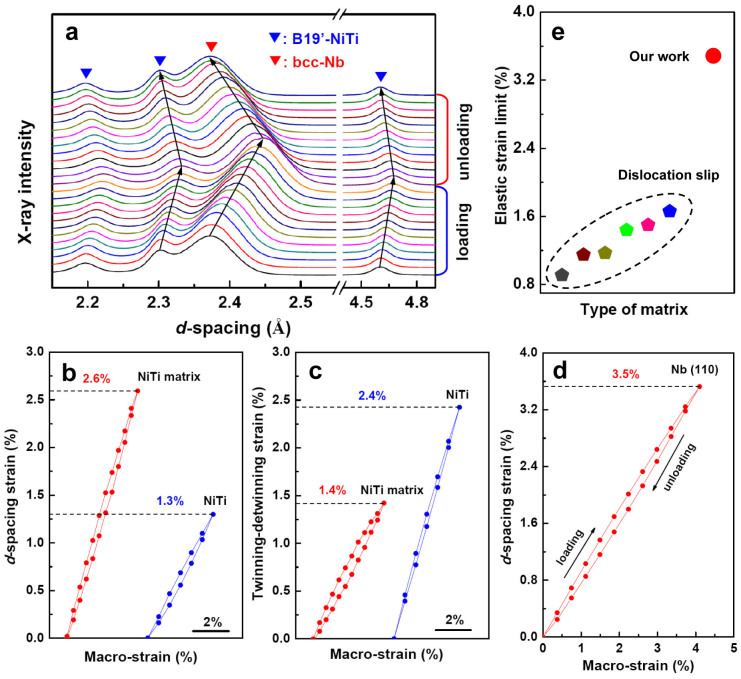
*In situ* synchrotron X-ray diffraction analysis of the NiTi-Nb composite and the binary NiTi alloy (with oriented martensite). (a), Evolution of *in situ* synchrotron X-ray diffraction patterns of the NiTi-Nb composite during a tensile deformation cycle to 4% macroscopic strain. (b), The *d*-spacing strain with respect to applied macroscopic strain for the B19′-NiTi (001) planes perpendicular to the loading direction in the NiTi matrix of the NiTi-Nb composite (the red curve) and in the binary NiTi alloy (the blue curve). (c), The calculated twinning-detwinning strains versus the applied macroscopic strain (the red curve for the NiTi matrix in the NiTi-Nb composite; the blue curve for the binary NiTi alloy). (d), The *d*-spacing strain with respect to applied macroscopic strain for the Nb (110) planes perpendicular to the loading direction in the NiTi-Nb composite. (e), Comparison of the elastic strain limits of the Nb nanowires embedded in oriented martensitic NiTi matrixand embedded in the conventional metal matrix deforming by dislocation slip^23^,^28^,^29^,^30^.

To reveal the mechanism of such superior mechanical properties of the composite, *in situ* synchrotron X-ray diffraction was carried out on the NiTi-Nb composite, and a binary NiTi alloy (with oriented martensite) for comparison, during tensile deformation cycle. Figure 4a shows the *in situ* synchrotron X-ray diffraction patterns of the composite obtained over a tensile deformation cycle up to 4% of strain. The *d*-spacing strain of the B19′ martensite in the loading direction, as determined from the B19′-NiTi (001) planes perpendicular to the loading direction, is plotted in Figure 4b as a function of the applied macroscopic strain (the red curve). It can be seen that the oriented martensitic NiTi matrix underwent a large tensile elastic strain of 2.6%. For comparison, the *d*-spacing strain of the B19′-NiTi (001) planes in the binary NiTi alloy (with oriented martensite) is also plotted in Figure 4b (the blue curve). It is seen that the binary NiTi alloy exhibited a tensile elastic strain of 1.3%, which is substantially smaller than that (2.6%) of the NiTi matrix in the NiTi-Nb composite. It is believed that the existence of large quantities of Nb nanowires can effectively refine grain sizes of matrix and introducelarge amounts of nanowire/matrix interfaces, which suppress the plastic deformation of the matrix and enhance the elastic deformation of the matrix.

The strains of the reversible twinning-detwinning deformations (*ε_t_*_−*d*_) of the matrix upon tensile cycling can be computed by the difference between the applied macroscopic strain and the elastic strain (*ε_e_*) of the matrix (*ε_t_*_−*d*_ = *ε_m_* − *ε_e_*). The calculated twinning-detwinning strains are shown in Figure 4c as a function of the applied macroscopic strain. It can be seen that the oriented martensitic NiTi matrix went through continuous reversible twinning-detwinning deformation throughout the tensile cycle. The reversible twinning-detwinning strains of the matrix are about 1.4% (red curve in Figure 4c), which is much smaller than those (3.7% − 1.3% = 2.4%) (blue curve in Figure 4c) of the binary NiTi alloy (with oriented martensite) in a tensile cycle of 3.7% (Figure S2). This result indicates that the reversible twinning-detwinning deformations in the matrix are significantly decreased due to the existence of Nb nanowires compared to the binary NiTi alloy (with oriented martensite).

Figure 4d shows the *d*-spacing strain with respect to applied macroscopic strain for the Nb (110) planes perpendicular to the loading direction. It is observed that the Nb nanowires embedded in the oriented martensitic NiTi matrix exhibit a large elastic strain of 3.5%, which is much larger than that (~1.5%)^23^,^29^,^30^,^31^ of the nanowires embedded in the conventional metal matrices deforming by dislocation slip (Figure 4e) and is comparable to that of freestanding nanowires^13^,^14^,^15^,^16^,^17^,^18^,^19^. This result demonstrates that the exceptional intrinsic mechanical properties of nanowires can be exploited in the oriented martensitic NiTi matrix deforming by elastic elongation and twinning-detwinning.

## Conclusions

In this study, we demonstrated that the exceptional intrinsic mechanical properties of nanowires can be exploited in the metal matrix deforming by elastic elongation and twinning-detwinning, and the mechanical properties of the matrix are greatly improved due to the existence of embedded nanowires. Stemming from the synergistic effect between nanowires and matrix, the *in-situ* Nb nanowires-NiTi matrix composite simultaneously possesses large linear elastic strain, high yield strength, high energy storage density and high energy storage efficiency. Given these superior properties, this material has great potential for many practical applications such as robotic actuators with accurate position feedback, strain seniors with high sensitivity, and mechanical energy storage and conversion devices with high efficiency in various fields. More broadly, this studyprovides new avenues for developing advanced composites with superior properties by using effective synergistic effect between components.

## Methods

An alloy ingot of 25 kilograms in weight with a composition of Ni_45_Ti_45_Nb_10_ (at.%) was prepared by vacuum induction melting. The raw materials used were of commercial purity Ni (99.96 wt.%), Ti (99.90 wt.%) and Nb (99.99 wt.%). The ingot was hot-forged at 850°C into a rod of 8 mm in diameter and further hot-drawn at 750°C into a thick wire of 1 mm in diameter. Then the hot-drawn wire was cold-drawn into thin wires of 0.5 mm in diameter at room temperature with no intermediate annealing. From the cold-drawn wire, the specimens with 10 cm in length were cut out and annealed at 450°C for 20 min followed by air cooling. The pre-deformation pretreatment (five times tensile strain cycles of 14%) was performed on the annealed specimen. After this pretreatment, the NiTi matrix with self-accommodated martensitic variants (many orientations) changed into the oriented martensitic NiTi matrix consisted of oriented martensitic variants.

In-situ synchrotron X-ray diffraction measurements were performed at the 11-ID-C beamline of the Advanced Photon Source at Argonne National Laboratory. High-energy X-rays of 115 keV energy and 0.6 mm × 0.6 mm beam size were used to obtain 2D diffraction patterns in the transmission geometry using a Perkin-Elmer large area detector placed at 1.8 m from the sample. The 2D diffraction patterns were collected during in-situ tensile deformation. In-situ tensile deformation was performed using an Instron testing machine at a strain rate of 5 × 10^−4^ s^−1^ and the total elongation of the gauge length was measured with a static axial clip-on extensometer. Microstructure of the composite wire was analyzed using a FEI Tecnai G2 F20 transmission electron microscope equipped with an energy dispersive X-ray spectroscopic analyzer operated at a voltage of 200 kV.

## Author Contributions

L.S.C. and S.J.H. designed the research topic of this project. Y.R. supervised the synchrotron experiments. S.J.H. and Y.R. carried out the synchrotron experiments. F.M.G. and X.B.S. carried out the material preparation and tensile experiments. S.J.H., L.S.C., Y.N.L., D.Q.J. and D.E.B. wrote the manuscript.

## Supplementary Material

Supplementary Information Supporting Information
