# Supplementary material for: Achieving large linear elasticity and high strength in bulk nanocompsite via synergistic effect
Source: Sci Rep. 2015 Mar 9;5:8892. doi: 10.1038/srep08892 (PMC4352871; doi:10.1038/srep08892)
Supplement: Supplementary Information — Supporting Information [file srep08892-s1.pdf]

Supporting Information

Achieving large linear elasticity and high strength in bulk nanocompsite via synergistic effect

Shijie Hao[1], Lishan Cui[1]*, Fangmin Guo[1], Yinong Liu[2], Xiaobin Shi[1], Daqiang Jiang[1], Dennis E Brown[3] & Yang Ren[4]*

1. State Key Laboratory of Heavy Oil Processing, China University of Petroleum, Beijing 102249, China
2. School of Mechanical and Chemical Engineering, The University of Western Australia, Crawley, WA 6009, Australia
3. Department of Physics, Northern Illinois University, De Kalb, Illinois 60115, USA
4. X-ray Science Division, Advanced Photon Source, Argonne National Laboratory, Argonne, Illinois 60439, USA

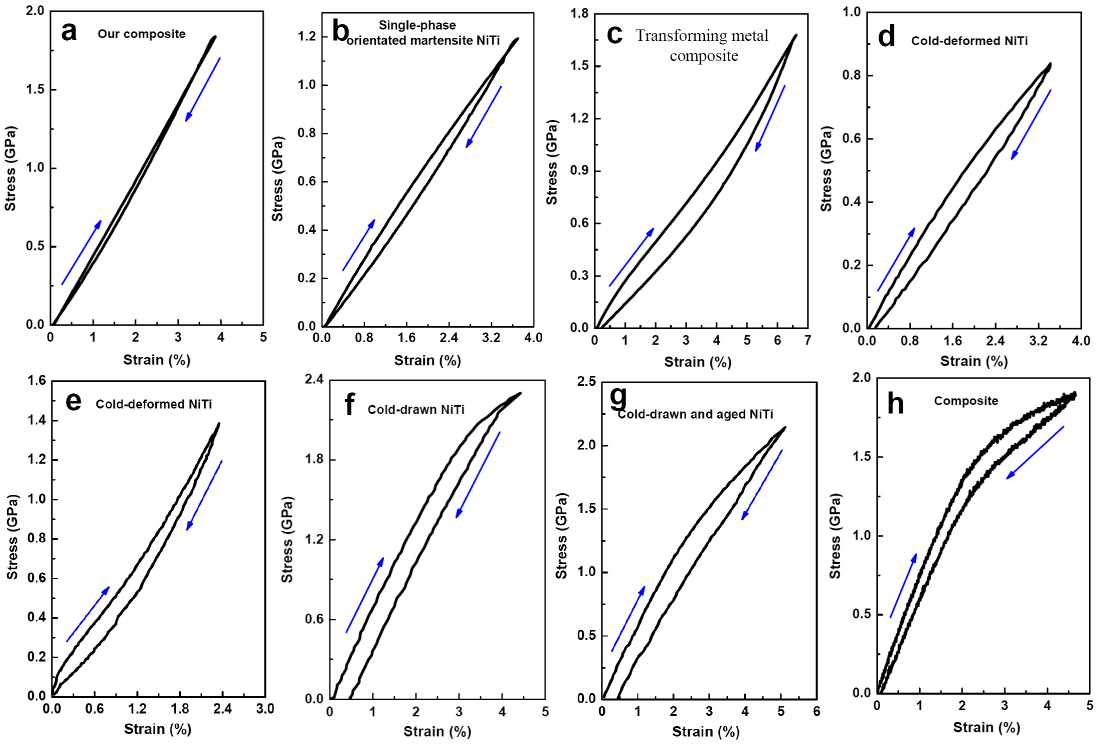

Figure S1 | Comparison of tensile stress-strain curves of our composite and the reported various metallic materials with large linear elasticity. Tensile stress-strain curves of our composite (a), the binary NiTi alloy wire (with oriented martensite) (Figure S2 for its fabrication details) (b), the transforming metal composite[23] (c), the 14% cold-drawn NiTi wire[7] (d), the plastically compressed by 11% NiTi specimen[9] (e), the 70% cold-drawn NiTi wire[10], the 70% cold-drawn and aged for 3.6 ks at 573 K NiTi wire[10] (g), the nanostructured Nb-NiTi composite[11] (h).

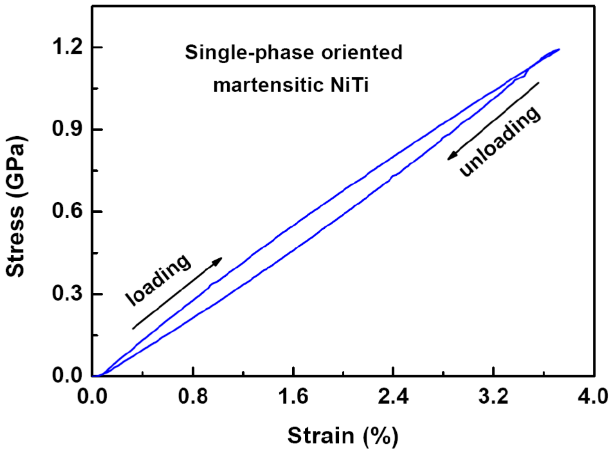

Figure S2 | Tensile stress-strain curve of the binary NiTi alloy wire (with oriented martensite). **Note:** The preparation process of the binary NiTi alloy wire with diameters of 0.5 mm is similar to that of the *in-situ* Nb nanowires - oriented martensite NiTi composite wire (refer to the Experimental Section).

Typical actuator (examples)	Strain (%)	Stress (MPa)	Energy efficiency (%)	Energy density (J/cm2)
Piezoelectric ceramics (PZT)	0.2	110	90	0.1
Electroactive polymer (silicone)	63	3	90	0.75
Megnetostrictive (Terfenol-D)	1	700	80	0.025
Spring steel	<1	1000	>90	<5
Shape memory alloy (NiTi)	5-9	600	<60	30-50
Orientated martensite NiTi	3.7	1200	82	23
Transforming metal composite	6.5	1650	82	52
Our composite (Nb-NiTi)	4	1800	96	36

Table S1 | Comparison of recoverable strain, yield stress, and mechanical energy storage density and storage efficiency between the *in-situ* Nb nanowires - oriented martensite NiTi composite and other various smart materials[11,21].
